# High Glucose Induces Sumoylation of Smad4 via SUMO2/3 in Mesangial Cells

**DOI:** 10.1155/2014/782625

**Published:** 2014-05-27

**Authors:** Xueqin Zhou, Chenlin Gao, Wei Huang, Maojun Yang, Guo Chen, Lan Jiang, Fang Gou, Hong Feng, Na Ai, Yong Xu

**Affiliations:** ^1^Department of Endocrinology, Affiliated Hospital of Luzhou Medical College, Luzhou, Sichuan 646000, China; ^2^Department of Endocrinology, The Central Hospital of Bazhong City, Sichuan 636000, China

## Abstract

Recent studies have shown that sumoylation is a posttranslational modification involved in regulation of the transforming growth factor-**β** (TGF-**β**) signaling pathway, which plays a critical role in renal fibrosis in diabetic nephropathy (DN). However, the role of sumoylation in the regulation of TGF-**β** signaling in DN is still unclear. In the present study, we investigated the expression of SUMO (SUMO1 and SUMO2/3) and Smad4 and the interaction between SUMO and Smad4 in cultured rat mesangial cells induced by high glucose. We found that SUMO1 and SUMO2/3 expression was significantly increased in the high glucose groups compared to the normal group (*P* < 0.05). Smad4 and fibronectin (FN) levels were also increased in the high glucose groups in a dose-dependent manner. Coimmunoprecipitation and confocal laser scanning revealed that Smad4 interacted and colocalized with SUMO2/3, but not with SUMO1 in mesangial cells. Sumoylation (SUMO2/3) of Smad4 under high glucose condition was strongly enhanced compared to normal control (*P* < 0.05). These results suggest that high glucose may activate TGF-**β**/Smad signaling through sumoylation of Samd4 by SUMO2/3 in mesangial cells.

## 1. Introduction

Diabetic nephropathy (DN) is a serious microvascular complication of diabetes and a leading cause of end-stage renal disease in developed countries. Early DN is characterized by mesangial expansion, thickened glomerular and tubular basement membranes, and accumulation of the extracellular matrix (ECM) and can progress to glomerulosclerosis and tubulointerstitial fibrosis in later stages [1–3]. At present, the pathogenesis of DN remains unclear, and then further study of molecular mechanisms to develop new treatment approaches for DN is required.

As a key mediator of fibrogenesis, transforming growth factor-*β* (TGF-*β*) plays a critical role in diabetic nephropathy, and Smad4 is a common mediator in TGF-*β* signaling [4–6]. The TGF-*β*/Smad pathway is modulated by several posttranslational modifications, including phosphorylation, ubiquitination, and acetylation [7, 8]. In a previous study, we demonstrated that ubiquitination of histones H2A and H2B is involved in diabetic nephropathy by activating the TGF-*β* signaling pathway [9]. Recent studies have demonstrated that sumoylation is a reversible posttranslational modification involved in regulation of the TGF-*β* signaling pathway [10, 11].

SUMO is a type of small ubiquitin-like molecule primarily involved in posttranslational modification of proteins, similar to ubiquitination. In mammals, there are four SUMO paralogues, SUMO1, SUMO2, SUMO3, and SUMO4 [12]. SUMO2 and SUMO3 share 95% homology with each other and are collectively referred to as SUMO2/3 [13]. Sumoylation is the covalent attachment of SUMO to specific target proteins via an ATP-dependent enzyme cascade, including E1 activating enzyme, E2 conjugating enzyme (Ubc9), and several E3 ligases [14]. Sumoylation plays an important role in multiple biological processes, such as protein interactions, protein stability, nuclear-cytoplasmic trafficking, transcriptional regulation, DNA repair, and cellular signaling pathways [15, 16]. Recent studies have shown that sumoylation regulates the TGF-*β* pathway by modifying several important signaling molecules, such as type I TGF-*β* receptor (T*β*RI) and Smad4 [17]. However, whether sumoylation is involved in the pathogenesis of DN is unknown.

In this study, we detected the levels of SUMO and Smad in each group to investigate whether they were regulated by high glucose and whether high glucose could induce sumoylation of Smad4 in rat mesangial cells. And we explored the role of sumoylation in regulating TGF-*β*/Smad signaling in DN.

## 2. Materials and Methods

### 2.1. Cell Culture

Rat glomerular mesangial cells (HBZY-1) were purchased from the Preservation Center at Wuhan University and cultured in low glucose DMEM (Hyclone) medium with 10% fetal bovine serum (FBS, Invitrogen) and 1% penicillin/streptomycin. Mesangial cells were cultured with serum free medium for 24 h before treatment, followed by addition of 5.6 mmol/L glucose as a normal control, 10, 20, and 30 mmol/L glucose as experimental groups, and mannitol as an osmotic control for 6 h, 12 h, and 24 h.

### 2.2. Cell Lysis and Western Blotting

Cells were washed three times with ice-cold phosphate-buffered saline (PBS) and lysed in lysis buffer (3.0 M NaCl, 1.0 M Tris PH 7.5, 1% TritonX-100, 10% SDS and protease inhibitor). Cell lysates were centrifuged at 4°C, 15,000 ×g for 10 min to pellet cell debris and protein samples were extracted. Supernatants were separated on SDS-PAGE and transferred onto PVDF membranes (Millipore). Membranes were blocked in 5% nonfat milk for 1 h and incubated with primary antibodies overnight at 4°C. Primary antibodies included mouse monoclonal anti-Smad4 (1 : 500, Santa Cruz), rabbit polyclonal anti-SUMO2/3 (1 : 600, Millipore), rabbit monoclonal anti-SUMO1 (1 : 800, Abcam), and mouse monoclonal anti-GAPDH (1 : 1,000, Beyotime Biotechnology). Membranes were incubated with horseradish peroxidase-linked anti-mouse and anti-rabbit secondary antibodies (Beyotime) at room temperature for 1 h. Proteins were detected using ECL reagents (Millipore) and the Bio-Rad chemiluminescence system and quantitatively analyzed by Quantity One software.

### 2.3. Immunoprecipitation and Immunoblotting

Cells were lysed in ice-cold IP lysis buffer (150 mM NaCl, 25 mM Tris PH 7.4, 1 mM EDTA, 1% NP-40, 5% glycerol and protease inhibitor). Cell lysates were centrifuged at 15,000 ×g at 4°C for 15 min, and the supernatants were subjected to immunoprecipitation with mouse anti-Smad4 antibody or mouse IgG as negative control overnight at 4°C. Immune complexes were precipitated with protein G agarose beads for 1-2 h under rotary agitation, and the agarose beads were washed four times in wash buffer. Bound proteins were eluted in 2x reducing sample buffer, and samples were analyzed by Western blotting as described using mouse anti-Smad4 antibody for Smad4 and rabbit anti-SUMO1 or SUMO2/3 antibody for SUMO-conjugated Smad4 protein.

### 2.4. Reverse Transcription-PCR

Cells were washed twice in ice-cold PBS and lysed in TRIzol reagent, followed by total RNA extraction using the RNAsimple Total RNA kit (Tiangen Biotech). Samples were reverse transcribed (RT) to cDNA using a kit according to the manufacturer's instructions (Bio-BRK). PCR was performed using the cDNA and the following primers: SUMO1, forward 5′-TATGGACAGGACAGCAG-3′, reverse 5′-CCATTCCCAGTTCTTTT G-3′; SUMO2/3, forward 5′-GACGAGAAACCCAAGGA-3′, reverse 5′-CTGCCGTTCACAATAG G-3′; FN, forward 5′-CAGCCTACGGATGAC TC-3′, reverse 5′-CTCTTTCTGCCACTGTTCT-3′; and GAPDH, forward 5′-GGTCATGAGTCCTTCCACGATA-3′, reverse 5′-ATGCTGGCGCTGAGTACGTC-3′. PCR products were analyzed using 2% agarose gels and visualized by a UV transilluminator.

### 2.5. Immunofluorescence

Mesangial cells were grown on coverslips in 6-well plates. Cells were fixed in 4% paraformaldehyde (Pierce) and permeabilized in 0.25% Triton X-100 (Sigma) for 10 min. Cells were washed twice in PBS and blocked in 5% goat serum for 1 h at room temperature, followed by incubation with anti-Smad4 and anti-SUMO2/3 antibodies overnight at 4°C. After washing, cells were incubated with rhodamine and fluorescein isothiocyanate-conjugated secondary antibodies (Bio-Synthesis) for 45 min in the dark. The coverslips were washed and mounted onto slides using mounting medium (Beyotime) and imaged with a DMIRE2 laser scanning confocal microscope (Leica, Germany).

### 2.6. Statistical Analyses

All data obtained from at least three independent experiments were expressed as the mean ± standard deviation (SD) and analyzed using one-way analysis of variance (ANOVA), followed by the LSD post hoc test for multiple comparisons (SPSS 11.5 statistical software). *P* < 0.05 was considered significant.

## 3. Results

### 3.1. High Glucose Induced SUMO1 and SUMO2/3 Expression in Mesangial Cells

**Figure 1:**
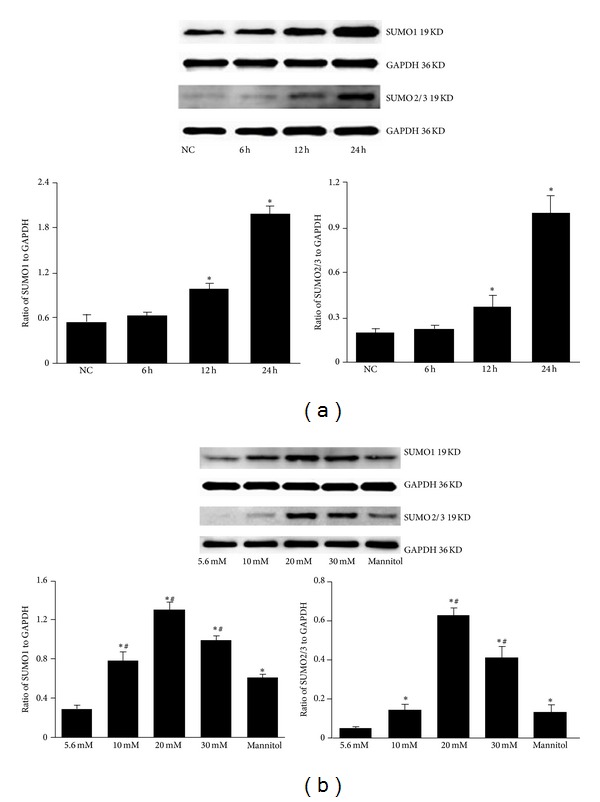
SUMO protein expression in mesangial cells detected by Western blot. (a) Cells were treated with 30 mmol/L high glucose for 6, 12, and 24 h, and Western blot was performed to detect SUMO1 and SUMO2/3 protein levels. *P < 0.05 compared to normal control (NC) group. (b) Cells were treated with the indicated concentrations of glucose or mannitol for 24 h, with 5.6 mmol/L glucose as a normal control and 30 mmol/L mannitol as an osmotic control. *P < 0.05 compared to normal control; ^#^ *P* < 0.05 compared to mannitol control. GAPDH was used as a loading control in (a) and (b).

**Figure 2:**
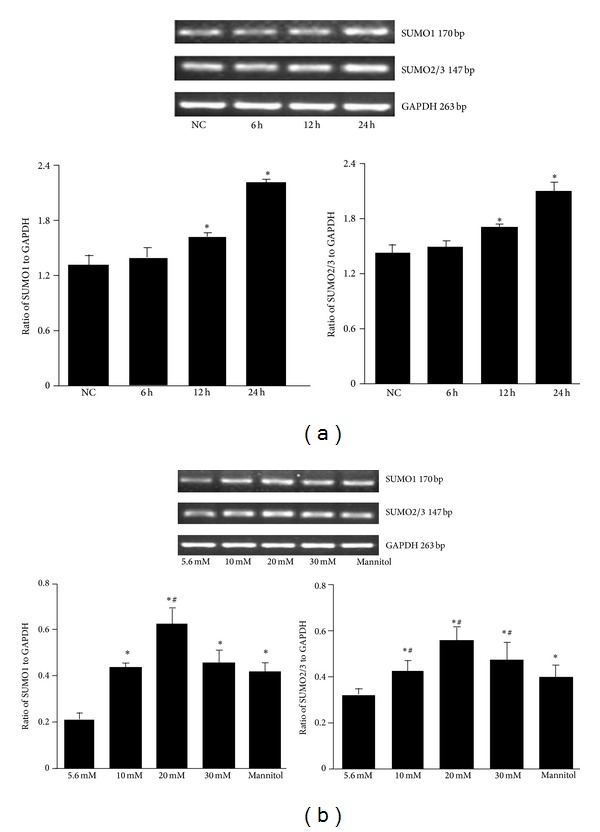
Reverse transcription-PCR analysis of SUMO mRNA expression. (a) Mesangial cells were treated with 30 mmol/L high glucose for 6, 12, and 24 h. SUMO1 and SUMO2/3 mRNA levels were assessed by RT-PCR and normalized to GAPDH. *P < 0.05 compared to normal control (NC) group. (b) Cells were treated with the indicated concentrations of glucose or mannitol for 24 h, with 5.6 mmol/L glucose as a normal control and 30 mmol/L mannitol as an osmotic control. GAPDH was used to confirm equal loading. *P < 0.05 compared to normal control; ^#^ *P* < 0.05 compared to mannitol control.

To determine whether SUMO is regulated by glucose in mesangial cells, we first detected SUMO proteins by Western blot. As shown in Figure 1(a) SUMO1 and SUMO2/3 expression in mesangial cells gradually increased after treatment with 30 mmol/L glucose for 12 h to 24 h and was highest at 24 h. There was no significant increase at 6 h compared to the normal control. SUMO1 and SUMO2/3 were highly expressed in the high glucose groups, particularly in the 20 mmol/L glucose group, compared to normal control (*P* < 0.05, Figure 1(b)). A significant difference was found between the mannitol and normal control groups. However, the expressions of SUMO1 and SUMO2/3 were significantly decreased in the mannitol group compared with the 20 mmol/L and 30 mmol/L glucose groups (*P* < 0.05), suggesting that osmotic pressure had a little effect on the high glucose-induced SUMO expression. Reverse transcription-PCR (RT-PCR) was performed to assess glucose-induced SUMO1 and SUMO2/3 mRNA expression. Results showed similar trend with SUMO protein expression (Figure 2).

### 3.2. High Glucose Induces Smad4 Expression in Mesangial Cells

**Figure 3:**
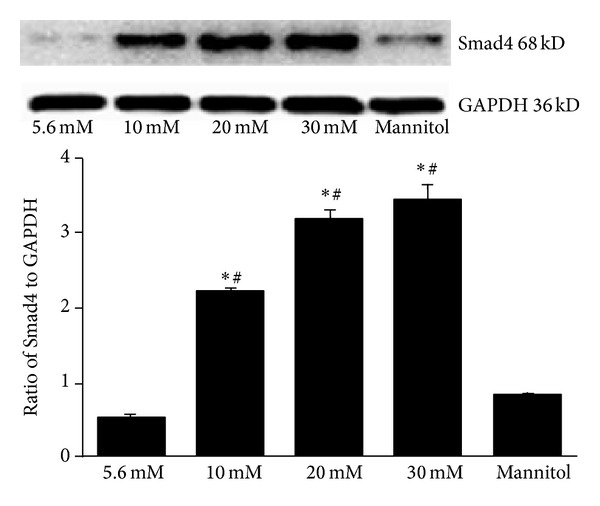
Smad4 protein expression in mesangial cells induced by high glucose. Mesangial cells were treated with the indicated concentrations of glucose or mannitol for 24 h, and Western blot was performed to detect Smad4 protein levels with 5.6 mmol/L glucose as a normal control and 30 mmol/L mannitol as an osmotic control. *P < 0.05 compared to normal control; ^#^ *P* < 0.05 compared to mannitol control.

Western blot was used to detect Smad4 protein levels in mesangial cells exposed to glucose or mannitol for 24 h. GAPDH was used as a loading control. As shown in Figure 3, Smad4 was abundantly expressed in the high glucose groups in a concentration dependent manner compared to normal control. In addition, there was no significant difference between the mannitol osmotic and normal controls, confirming that glucose, but not osmotic stress, increased Smad4 expression in mesangial cells.

### 3.3. Smad4 is Sumoylated by SUMO2/3 in Mesangial Cells

**Figure 4:**
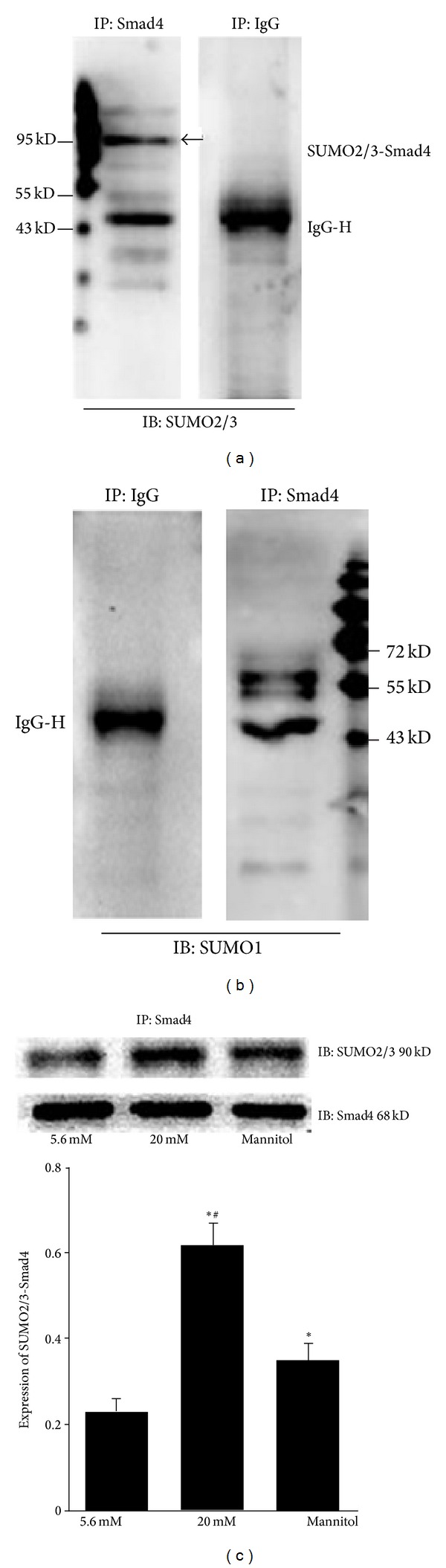
Smad4 is sumoylated by SUMO2/3 in mesangial cells. (a) and (b) Cell lysates were subjected to immunoprecipitation (IP) with anti-Smad4 antibody or normal mouse IgG as a negative control, followed by immunoblot (IB) with anti-SUMO2/3 or anti-SUMO1 antibody to detect the interaction between SUMO2/3 or SUMO1 with Smad4. The arrow marked band indicates SUMO2/3-Smad4 conjugates (a). No specific band was detected for SUMO1-Smad4 conjugates in mesangial cells (b). IgG-H marks the IgG heavy chain in (a) and (b). (c) Cells were treated with 5.6 mmol/L and 20 mmol/L glucose and equimolar concentration of mannitol for 24 h. Anti-Smad4 immunoprecipitates were subjected to immunoblotting with anti-Smad4 or anti-SUMO2/3 antibodies for Smad4 and SUMO2/3-Smad4 proteins. Relative expression of SUMO2/3-Smad4 conjugates was detected by Western blot and normalized to Smad4. *P < 0.05 compared to normal control; ^#^ *P* < 0.05 compared to mannitol control.

To determine whether Smad4 is sumoylated in mesangial cells, cell lysates were subjected to immunoprecipitation with anti-Smad4 antibody. Normal mouse IgG was used as a control antibody and immunoprecipitates were immunoblotted with anti-SUMO2/3 or anti-SUMO1 antibodies. An approximately 90 kDa band representing SUMO2/3-Smad4 conjugates was detected, and its size was compatible with the addition of a single SUMO2/3 molecule to Smad4 (Figure 4(a)). In contrast, the mouse IgG control antibody did not detect sumoylated Smad4 protein.  Figure 4(b) shows that a specific band for SUMO1-Smad4 conjugated protein (about 90 kDa) was not detected, indicating that Smad4 is not sumoylated by SUMO1 in mesangial cells. To further assess high glucose-induced sumoylation of Smad4, anti-Smad4 immunoprecipitates were immunoblotted with anti-Smad4 or anti-SUMO2/3 antibodies after treating mesangial cells with various concentrations of glucose or mannitol for 24 h. As shown in Figure 4(c), a SUMO2/3-Smad4 conjugated protein was strongly expressed in the 20 mmol/L glucose group compared to normal and mannitol controls (*P* < 0.01). These results suggest that high glucose but not osmotic stress activates sumoylation of Smad4 via SUMO2/3 in mesangial cells.

### 3.4. Colocalization of SUMO2/3 and Smad4 in Mesangial Cells

**Figure 5:**
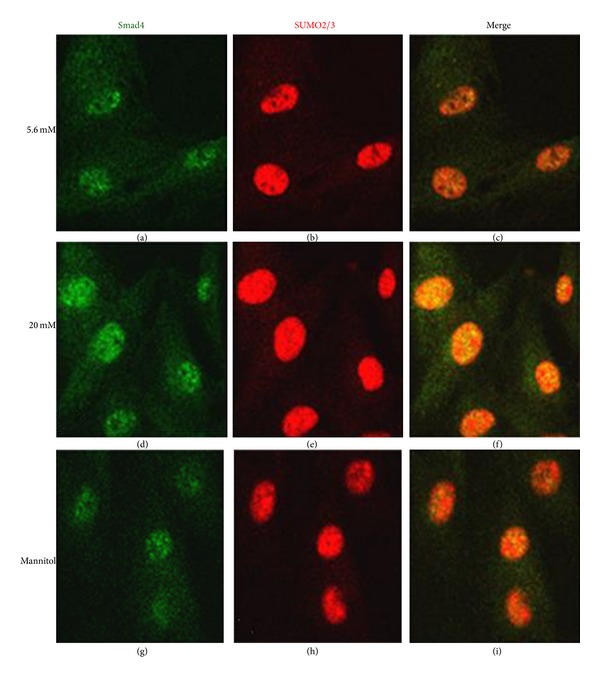
Colocalization of SUMO2/3 and Smad4 in mesangial cells. Cells were incubated with mouse anti-Smad4 monoclonal antibody and rabbit anti-SUMO2/3 polyclonal antibody and visualized with fluorescein isothiocyanate-conjugated anti-mouse IgG for Smad4 ((a), (d), (g)) and rhodamine-conjugated anti-rabbit IgG for SUMO2/3 ((b), (e), (h)). The merged images of Smad4 and SUMO2/3 stainings in each group are shown in (c), (f), and (i).

Immunofluorescence was performed to determine subcellular localization of SUMO2/3 and Smad4 after mesangial cells were treated with high glucose for 24 h. Cells were incubated with anti-Smad4 and anti-SUMO2/3 primary antibodies and stained with fluorescein isothiocyanate and rhodamine-conjugated secondary antibodies. Under confocal immunofluorescence microscopy, Smad4 showed diffuse cytoplasmic and nuclear distribution (Figures 5(a), 5(d), and 5(g)), while SUMO2/3 was localized primarily in the nucleus with a punctated pattern in each group (Figures 5(b), 5(e), and 5(h)). The merged images showed that SUMO2/3 colocalized with Smad4 primarily in the nucleus and was strongly enhanced in the high glucose group (Figure 5(f)) compared with normal (Figure 5(c)) and mannitol controls (Figure 5(i)).

### 3.5. High Glucose Induced Fibronectin (FN) mRNA Expression in Mesangial Cells

**Figure 6:**
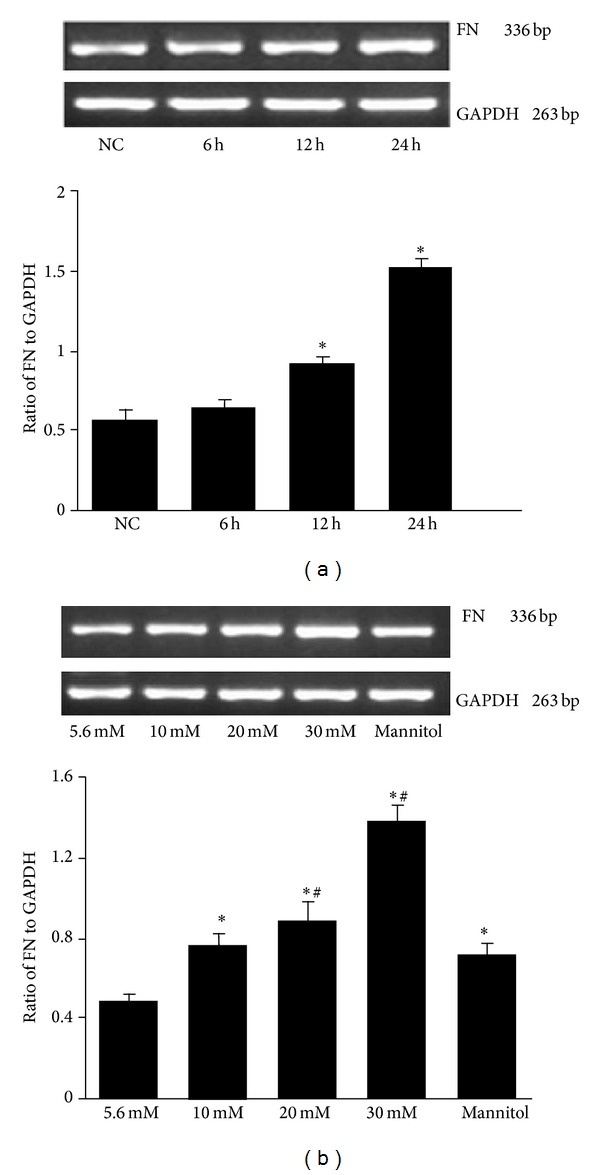
Reverse transcription-PCR analysis of FN mRNA expression in mesangial cells. (a) Mesangial cells were treated with 30 mmol/L high glucose for 6, 12, and 24 h, and FN mRNA expression levels were detected by RT-PCR. *P < 0.05 compared to normal control (NC) group. (b) Cells were treated with different concentrations of glucose or mannitol for 24 h, with 5.6 mmol/L glucose as a normal control and 30 mmol/L mannitol as an osmotic control. *P < 0.05 compared to normal control; ^#^ *P* < 0.05 compared to mannitol control.

Fibronectin (FN) is an ECM protein in diabetic kidney. RT-PCR analysis of FN mRNA in mesangial cells showed that the expression of FN mRNA increased in high glucose groups compared to the normal control group (*P* < 0.05) in a concentration and time dependent manner (Figure 6). A significant difference between the mannitol and normal control groups was observed. However, the expression of FN mRNA was lower in the mannitol group compared with the 20 mmol/L and 30 mmol/L glucose groups (*P* < 0.05; Figure 6(b)), indicating that high glucose-induced the increase of FN mRNA expression was not an osmotic effect.

## 4. Discussion

TGF-*β* has been considered a key mediator in multiple organ fibrosis, including the heart, liver, lung, and kidney [18–20]. TGF-*β* signaling is transduced by transmembrane receptors (T*β*RII, T*β*RI) and intracellular signals called Smads (Smad1-8) [21]. Hyperglycemia, AGE, and angiotensin II have been shown to activate TGF-*β* signaling in mesangial and tubular cells [5, 6]. Diabetic glomerulosclerosis and tubulointerstitial fibrosis are caused by accumulation of ECM proteins and glomerular basement membrane thickening [22, 23]. FN is one of the major components of the ECM and is useful in evaluating pathological conditions in DN [24]. Our data show that high glucose increased the expression of FN in mesangial cells, further confirming that high glucose promotes the fibrosis in DN.

Many studies have indicated that phosphorylation and activation of Smad2/3 contribute to diabetic renal fibrosis [25, 26]. However, the role of Smad4 in TGF-*β*-mediated renal fibrosis is largely unclear. Therefore, we investigated whether Smad4 is regulated by high glucose in cultured mesangial cells. As expected, our results showed that high glucose increased Smad4 expression in a concentration dependent manner, indicating that Smad4 is positively involved in TGF-*β*-mediated progression of DN. This finding is similar to that of Meng et al. [27], who revealed that deletion of Smad4 inhibited progression of renal fibrosis.

Our previous study showed that ubiquitination of histones H2A and H2B could activate the TGF-*β* signaling pathway in diabetic nephropathy [9]. However, several studies have suggested that use of the specific ubiquitin proteasome inhibitor MG132 cannot completely block activation of the TGF-*β* signaling pathway [28, 29]. This indicates that other protein modifications are also involved in TGF-*β* pathway activation in DN. For example, sumoylation has been demonstrated to play an important role in a wide range of biological processes and several human diseases, such as inflammation, cancer, heart disease, and neurodegenerative diseases [30–33]. However, it is not known whether sumoylation is involved in DN.

SUMO1 and SUMO2/3 are expressed in most tissues, whereas SUMO4 expression appears to be limited to the kidney, lymph node, and spleen [34]. In addition, various cellular stresses, such as osmotic, oxidative stress, and heat shock, have been shown to increase sumoylation by SUMO2/3 but have little effect on SUMO1 modification. Interestingly, a previous study reported that M55V substitution in the SUMO4 gene (163A→G) was strongly associated with type 1 diabetes [35]. To determine whether SUMOs are expressed and regulated by glucose in mesangial cells, we used Western blot and RT-PCR for detection. Our data show that SUMO1 and SUMO2/3 are expressed in mesangial cells, and high glucose increased the expression of SUMO. Meanwhile, we found that osmotic stress had little effect on the expression of SUMO1 and SUMO2/3 compared with high glucose. These results suggest that SUMO1 and SUMO2/3 may be involved in the progression of DN. The detailed mechanisms involved will require further study.

More than 120 SUMO substrate proteins have been identified. The majority are nuclear proteins, and most of nonnuclear substrates are signal transduction proteins, including NEMO, I*κ*B*α*, Smads, Glut4, and T*β*RI [36, 37]. Sumoylation changes the activity, subcellular localization, or stability of these proteins to influence signal transduction [15]. Sumoylation has been demonstrated to regulate the canonical TGF-*β*/Smad pathway through T*β*RI, Smad3, and Smad4 [10, 11, 38]. Several studies have shown that Smad4 is sumoylated by SUMO1 or SUMO2/3. Interestingly, these findings indicate an opposite effect of sumoylation on Smad4 activity, which can play a positive [39–41] or negative [42] role in regulating TGF-*β*/Smad signaling.

Given the important role of sumoylation in the TGF-*β*/Smad pathway and previously described results, we hypothesized that sumoylation of Smad4 may have a role in TGF-*β*-mediated DN. Therefore, we determined whether Smad4 is sumoylated and the effect of high glucose on covalent modification. As expected, our data showed that Smad4 interacted and colocalized with SUMO2/3 in mesangial cells and the expression was strongly enhanced by glucose, and osmotic stress has been shown to have little effect on sumoylation by SUMO2/3. Unfortunately, we found that Smad4 is not sumoylated by SUMO1 in mesangial cells. The results suggest that high glucose stimulates sumoylation of Smad4 through SUMO2/3 but not SUMO1 in mesangial cells, resulting in activation of the TGF-*β* signaling pathway. The mechanisms involved may include the sumoylation of Smad4, preventing the degradation of Smad4, increasing the level of Smad4, and enhancing nuclear accumulation and stability of Smad4. We did not analyze other regulators, such as several R-Smads and TGF-*β* receptors in this study. Additional studies are necessary to explore the diverse roles of sumoylation in TGF-*β*/Smad signaling, which mediates pathogenesis of DN.

A recent in vivo study first reported that inhibition of sumoylation by knockdown of Ubc9 suppresses canonical TGF-*β*/Smad signaling and prevents development of fibrosis in systemic sclerosis [43]. Netherton and Bonni reported that sumoylation plays an important role in TGF*β*-induced epithelial mesenchymal transition (EMT), which contributes to fibrotic and neoplastic diseases [44]. Moreover, another study demonstrated that sumoylation was involved in the progression of glomerulonephritis by regulating *α*-smooth muscle actin transcription in mesangial cells [45]. All of these findings suggest that sumoylation may be a new therapeutic target for fibrotic diseases and is likely to provide new insights into the pathogenesis of these diseases.

In summary, our study found that high glucose significantly increased the expression of SUMO1 and SUMO2/3 and stimulated Smad4 sumoylation through SUMO2/3 but not SUMO1. We demonstrated that sumoylation of Smad4 may be involved in the pathogenesis of DN by regulating the TGF-*β*/Smad signaling pathway. Further studies on sumoylation will be necessary to find potential therapeutic targets for DN.
